# Experiences of peer navigators implementing a bilingual multilevel intervention to address sexually transmitted infection and HIV disparities and social determinants of health

**DOI:** 10.1111/hex.13698

**Published:** 2023-01-04

**Authors:** José A. Robles Arvizu, Lilli Mann‐Jackson, Jorge Alonzo, Manuel Garcia, Lucero Refugio Aviles, Benjamin D. Smart, Scott D. Rhodes

**Affiliations:** ^1^ Department of Social Sciences and Health Policy Wake Forest University School of Medicine North Carolina Winston‐Salem USA

**Keywords:** communities of colour, LGBTQ, sexual health, social determinants of health, youth

## Abstract

**Introduction:**

Sexually transmitted infections (STIs) and human immunodeficiency virus (HIV) disproportionately affect young gay, bisexual and other men who have sex with men (GBMSM) and transgender women of colour. We explored the experiences of community‐based peer navigators (‘Community Navigators’) who participated in *Impact Triad*, a bilingual multilevel intervention developed by our community‐based participatory research partnership to reduce STIs and HIV and address social determinants of health (e.g., employment, education, social support and discrimination) among young GBMSM and transgender women of colour.

**Methods:**

Individual in‐depth interviews were conducted with 15 Community Navigators who participated in *Impact Triad*. Themes were identified through constant comparison.

**Results:**

Community Navigators' mean age was 31.4 years. Seven were self‐identified as African American/Black, 5 as Latine, 2 as multiracial/multiethnic, 1 as Asian American, 10 as cisgender men, 4 as transgender women and 1 as gender nonbinary. Thirteen themes emerged in three domains: (1) key aspects of the Community Navigator role (e.g., desire to serve as a community resource, the importance of being part of the communities in which one was working, the value of having an official role, being connected to other Community Navigators to problem‐solving and sustaining intervention aspects long‐term); (2) experiences implementing *Impact Triad* (e.g., engaging community members, meeting prioritized needs, building trust, using social media, increasing awareness and knowledge and challenges related to COVID‐19) and (3) lessons learned for future interventions (e.g., facilitating access to broader resources, building additional skills and increasing interactions among Community Navigators).

**Conclusion:**

Interviews identified important learnings about serving as Community Navigators and implementing *Impact Triad* that can guide future efforts to address STI/HIV disparities and social determinants of health through community‐based peer navigation.

**Patient or Public Contribution:**

Throughout this intervention trial, our partnership worked collaboratively with a study‐specific community advisory board (CAB) comprised primarily of young GBMSM and transgender women of colour. Members of this CAB participated in all aspects of the trial including trial design, intervention development, recruitment and retention strategies, data collection and analysis, interpretation of findings and dissemination.

## 1 INTRODUCTION

### 1.1 Sexually transmitted infection (STI) and human immunodeficiency virus (HIV) disparities

There is an urgent need to address disparities related to STIs and HIV in the United States. STI and HIV rates are highest among young persons,^1^, ^2^ and gay, bisexual and other men who have sex with men (GBMSM) and transgender women, and particularly those who are persons of colour, also carry a disproportionate burden of STIs and HIV. For example, GBMSM comprise approximately 4% of the US adult male population but 43% of all syphilis cases in the country and 86% of all HIV diagnoses among men,^2^, ^3^, ^4^ and if current rates persist, one in two African American/Black and one in four Latine GBMSM may be diagnosed with HIV during his lifetime.^5^ (The term ‘Latine’ uses a gender‐neutral ‘e’, which replaces the gendered endings ‘a’ and ‘o’ as in ‘Latina’ and ‘Latino’, and is similar to ‘Latinx’; this term is increasingly used within Latine LGBTQ communities). It is estimated that 14% of transgender women in the country are living with HIV, with prevalence rates as high as 44% among African American/Black and 26% among Latine transgender women.^6^ Furthermore, the US South has high STI rates compared to other regions of the country^1^, ^7^ and has been referred to as the ‘new’ and ‘latest’ US HIV epicentre.^8^

### 1.2 Social determinants of health

The issues contributing to STI and HIV disparities experienced by young GBMSM and transgender women of colour are complex. Social determinants that influence the health of these communities include individual, sociocultural, environmental, system and policy factors. For example, GBMSM and transgender women, particularly those who are persons of colour, are more likely than heterosexual and cisgender counterparts to experience limited access to employment and education and face high rates of discrimination in a range of settings including health care, workplaces and schools.^9^, ^10^, ^11^, ^12^ In addition, employment status, education level and related factors such as poverty, income and health insurance coverage have been associated with STI and HIV incidence and outcomes.^13^, ^14^ There has been a call to broaden the focus of STI and HIV prevention efforts to address these ‘upstream’ factors that profoundly impact sexual health, in addition to individual‐level factors such as increasing correct and consistent condom use and STI and HIV screening.^15^, ^16^

### 1.3 Community‐based participatory research (CBPR) and community‐based peer navigation

CBPR, which engages community members and community organizations as partners in all phases of the research process,^17^ has been identified as an important approach to understanding and reducing disproportionate STI and HIV burdens.^18^, ^19^ Community‐based peer navigation leverages natural helping within existing social networks and has been used in STI and HIV prevention efforts with promising results.^20^, ^21^, ^22^, ^23^, ^24^ There is a need for further development and evaluation of community‐based peer navigation interventions to address STIs and HIV as well as contributing social determinants using CBPR.

### 1.4 Centers for Disease Control and Prevention (CDC) Community Approaches to Reducing Sexually Transmitted Diseases (CARS) initiative and the *Impact Triad* intervention

CARS is a unique initiative of the US CDC that promotes the use of community engagement to increase STI and HIV prevention, screening and treatment and address related social determinants of health within communities disproportionately affected by STIs and HIV. The initiative focuses on identifying and implementing innovative community‐driven strategies that leverage community assets.^25^ As part of the CARS initiative, our CBPR partnership developed and tested *Impact Triad*, a bilingual (English and Spanish), multilevel intervention designed to reduce STIs and HIV and improve social determinants of health among young GBMSM and transgender women of colour in a high‐incidence community within the US South.

### 1.5 Purpose

A better understanding of the implementation of community‐based peer navigation interventions is essential to strengthening future STI and HIV prevention efforts among communities facing health disparities. As a component of the process evaluation of *Impact Triad*, we qualitatively explored the experiences of community‐based peer navigators within the intervention, using individual in‐depth interviews.

## 2 METHODS

### 2.1 Development of *Impact Triad*

The *Impact Triad* intervention trial was conducted by the North Carolina Community Research Partnership. This partnership is a long‐standing CBPR partnership comprised of community members, community organization and clinic representatives and academic researchers.^18^ Throughout this trial, our partnership worked collaboratively with a 15‐member community advisory board (CAB) comprised primarily of young GBMSM and transgender women of colour.

We first conducted a community‐driven needs assessment to identify and better understand needs and priorities related to STI and HIV prevention, screening and treatment and social determinants of health among young GBMSM and transgender women of colour locally, as well as existing community assets. Through this process, the CAB and CBPR partnership prioritized four social determinants of health as particularly salient for young GBMSM and transgender women of colour and their risk for STIs and HIV: employment, education, social support and discrimination. Based on needs assessment findings, the CAB and CBPR partnership developed intervention strategies to reduce STIs and HIV and improve these social determinants of health and integrated these strategies into the *Impact Triad* intervention, the details of which are described elsewhere.^26^

Briefly, *Impact Triad* includes three primary multilevel strategies: community‐based peer navigation, use of social media and antidiscrimination trainings for community organization staff. The intervention involved training young GBMSM and transgender women of colour as community‐based peer navigators, known as ‘Community Navigators’ in English and ‘Navegantes Comunitarios’ in Spanish, to carry out helping activities with members of their social networks in the community within the context of their daily lives. In addition to one‐on‐one and group‐level in‐person helping to share information and resources related to STI and HIV prevention, screening and treatment and prioritized social determinants of health, Community Navigators created and updated intervention‐related social media accounts (e.g., Facebook and Instagram) and used their own social media accounts for messaging with social network members. Community Navigators were also involved in the development of brief online video testimonials designed to raise consciousness among community organizations about the challenges and barriers young GBMSM and transgender women of colour face when accessing services related to STIs and HIV and social determinants of health and ways to facilitate access (https://www.youtube.com/channel/UCd7gOGhBerT0w1CTq5BwMcQ).

### 2.2 Community Navigator recruitment and training

Fifteen young GBMSM and transgender women of colour identified as informal leaders among their communities were recruited and trained to serve as *Impact Triad* Community Navigators. To be eligible to serve as a Community Navigator, a participant was 18 years of age or older; self‐identified as a person of colour; self‐identified as a man or as a transgender woman; reported sex with men and provided informed consent.

Community Navigators completed four 4‐h training sessions, in two cohorts of seven to eight Community Navigators each. They were trained to serve as (1) health advisors to provide information to social network members to meet needs and priorities related to STI and HIV prevention (e.g., condom and pre‐exposure prophylaxis [PrEP] access and use), screening and treatment, as well as social determinants of health (e.g., employment and education), offering guidance on where and how to access available services; (2) opinion leaders to bolster healthy and reframe unhealthy norms and expectations related to STIs and HIV or social determinants of health (e.g., social support) and (3) community advocates to bring the voices of young GBMSM and transgender women of colour to local community organizations by sharing feedback for improvement based on the perspectives of social network members. Community Navigators were trained to use an adapted version of the ‘ask‐advise‐assist’ model,^27^ represented by the acronym ‘IMPACT’ in English or ‘IMPACTO’ in Spanish with each letter representing a step in the natural helping process. A low‐literacy wallet‐sized reminder card was developed to serve as a ‘cheat sheet’ for Community Navigators outlining these steps to support others.^26^ Upon graduation, Community Navigators received a framed certificate of training completion, an identification badge, a t‐shirt and bag with the *Impact Triad* intervention logo and intervention materials (e.g., condoms, penis models and informational brochures) to carry out their work within their social networks and communities of young GBMSM and transgender women of colour. Community Navigators worked informally and formally with members of their social networks for 12 months and met monthly as a group with one another and with members of the CBPR partnership in convenient locations within trusted community organizations to plan, coordinate and evaluate their activities.

Community Navigators were provided a $50 stipend for each training session and each month of the 12‐month intervention implementation. They also were provided $50 per month to compensate for transportation costs.

### 2.3 Qualitative interviews

After the conclusion of intervention implementation, individual in‐depth interviews were conducted with all 15 *Impact Triad* Community Navigators. Standardized interview guides were developed in English and Spanish with careful consideration of wording, sequence and content to explore experiences with the intervention and current issues affecting the health of young GBMSM and transgender women of colour. Abbreviated sample items from the guide are outlined in Table 1.

**Table 1:** In‐depth interview guide with domains and abbreviated sample items

*Impact Triad* project
Tell me about how you became involved with the *Impact Triad* project.
When you became a Community Navigator, was your role how you thought it would be? What was different than you expected?
What did you like about being a Community Navigator?
What was hard about being a Community Navigator?
Training
How well prepared were you to serve as a Community Navigator? What other training would have helped you?
What suggestions for other topics should we have included?
How new for you was the information you received?
Recruitment
Tell me how you recruited the young gay and bisexual men and transgender women of colour who are part of your social network to participate in *Impact Triad*.
Community Navigator experience
What are some challenges that you faced as you began working with other young gay and bisexual men and transgender women of colour in your community?
What were some of the things that made it easier to talk to other young gay and bisexual men and transgender women of colour about sexual health? To talk about social determinants of health, such as employment, education, social support and discrimination? What were some of the things that made it difficult?
What were the most popular topics that young gay and bisexual men and transgender women wanted to discuss? What topics did they not seem comfortable talking about?
How did you use social media to work with young gay and bisexual men and transgender women of colour?
What do you think worked well with social media?
What challenges did you have using social media? Were you able to work them out?
Think back to before *Impact Triad* started. Are the topics that you and your friends talk about now any different? How have the ways your friends provide support to one another changed?
How were the monthly meetings with the *Impact Triad* coordinators and the other Community Navigators?
PrEP
How do the people who were part of your group feel about PrEP?
How interested are they in PrEP?
What barriers do they face to using PrEP?
Alcohol and drug use
How common is alcohol use among the people who were part of your group? Among other young gay and bisexual men and transgender women of colour?
How about drug use? What types of drugs do they use?
How common is it to use alcohol when having sex?
How common is it to use drugs when having sex? What drugs are used during sex?
What does alcohol and drug use have to do with risky sexual behaviours?
Social determinants of health
In *Impact Triad*, we focused on employment, education, social support and discrimination as social determinants of health. What else affects the health and well‐being of young gay and bisexual men and transgender women of colour?
How big of an issue is housing for young gay and bisexual men and transgender women of colour? How big of an issue is access to food? How do these issues affect their health?
Individual perceptions and empowerment
What do you think healthy sexuality means to young gay and bisexual men and transgender women of colour in our local community? Have your ideas of what healthy sexuality looks like changed since participating in *Impact Triad?*
What do you think would be good ideas or ways to prevent STIs and HIV among young gay and bisexual men and transgender women of colour living in your community? What do you think would be good ideas or ways to improve social determinants of health?
Do you think you will continue your work as a Community Navigator? Why?

Because of the COVID‐19 pandemic, interviews were conducted via telephone. Two trained bilingual members of our CBPR partnership conducted the interviews in English (*n* = 12) and Spanish (*n* = 3). Interviews averaged 45 min in length. All Community Navigators provided written consent and were compensated $50 for participating in an interview. Human subject protection and oversight were provided by the Wake Forest University School of Medicine Institutional Review Board.

### 2.4 Data analysis and interpretation

All interviews were digitally recorded and transcribed. Constant comparison, an approach to grounded theory, was used to analyse interview data. Constant comparison combines qualitative coding with simultaneous comparison; initial observations are continually refined throughout data collection and analysis.^28^ Three analysts read and reread interview transcripts, compared and contrasted content categories based on each analyst's interpretation of the data and identified emerging themes. After preliminary themes were developed, analysts came together with other CBPR partnership members via WebEx (a videoconferencing platform) in multiple meetings to discuss and reconcile final themes using an iterative process. Themes were also presented to the CAB in a meeting via WebEx for refinement and validation; CAB members were invited to respond to the themes and offered insights regarding themes that resonated with their own experiences and themes that they considered priorities for informing future efforts to improve the health of young GBMSM and transgender women of colour. At each stage in the analysis and interpretation process, a consensus approach was used, resolving discrepancies through discussion.

## 3 RESULTS

### 3.1 Community Navigator characteristics

Key characteristics of the *Impact Triad* Community Navigators at the time of enrolment in the intervention trial are presented in Table 2. The average Community Navigator age was 31.4 years. Seven Community Navigators (47%) identified as African American/Black, 5 as Latine (33%), 2 as multiracial/multiethnic (13%) and 1 as Asian American (7%); 10 identified as cisgender men (67%), 4 as transgender women (27%) and 1 as gender nonbinary (7%); 10 identified as gay (67%), 4 as heterosexual (27%) and 1 as bisexual (7%); 4 had a high school diploma or lower (27%) and 11 had at least some college (73%); 5 were in school full‐time (33%) and 12 were employed (80%).

**Table 2:** Select characteristics of *Impact Triad* Community Navigators (*n* = 15)

Characteristic	Mean (SD) or *n* (%)
Age (years)	31.4 (8.4)
Racial/ethnic identity	
African American/Black	7 (47)
Hispanic/Latine	5 (33)
Multiracial/multiethnic	2 (13)
Asian American	1 (7)
Gender	
Cisgender man	10 (67)
Transgender woman	4 (27)
Gender nonbinary	1 (7)
Sexual orientation	
Gay	10 (67)
Heterosexual	4 (27)
Bisexual	1 (7)
Education level	
Less than a high school diploma or equivalent	1 (7)
High school diploma or equivalent	3 (20)
Some college	5 (33)
2‐year degree	1 (7)
4‐year degree	4 (27)
Master's degree, professional degree or more	1 (7)
Student status	
Not in school	10 (67)
In school full‐time	5 (33)
Employment status	
Employed	12 (80)
Disabled and not working	2 (13)
Unemployed	1 (7)

### 3.2 Qualitative findings

Thirteen themes emerged from the interviews and were organized into three domains: (1) key aspects of the Community Navigator role, (2) experiences implementing the *Impact Triad* intervention and (3) lessons learned for future interventions.

#### 3.2.1 Key aspects of the Community Navigator role

Community Navigators identified several important aspects of fulfilling their role within *Impact Triad*. These themes, along with selected quotations, are presented in Table 3.

**Table 3:** Key aspects of the Community Navigator role (qualitative themes with select quotations from in‐depth interviews)

Desire to serve as a community leader
Community Navigators valued serving as leaders and as resources to address social determinants of health within their communities of identity.
*I liked getting to know other Latinos, and that I learned more about STDs and helped the community to find resources that helped them to live better*. (Participant [P] 15)
*I am educated and went to college, but I never felt like I achieved anything. After being a Community Navigator I feel like I have something to give to the community and it's great*. (P11)
Importance of being part of the communities in which one was working
Being members of the communities in which they worked was fundamental to trust building and successful intervention implementation by Community Navigators.
*We keep it one hundred, real, and hit the street, because that is what people respect and is how we earn trust, because we are demonstrating how we are part of this world*. (P12)
*Yo creo que mi credibilidad—ellos creen en mí—y que soy parte de la comunidad, eso fue lo que me ayudó. [I believe that my credibility—they put their faith in me—and that I am part of the community, that is what helped me out.]* (P2)
Value of having an official role
Having official titles and roles contributed to Community Navigators' self‐assurance and validated their work in communities.
*Podíamos hablar de temas de sexualidad sin que fuera tabú. Y era más fácil teniendo el respaldo de una institución y un programa para resolver algunas de las preguntas que tenían algunos de mis amigos y les daba pena hacer. [We were able to discuss topics about sexuality without it being taboo. It was also easier having the backing of an institution and program to assist in answering the questions that some of my friends had but were shy to ask.]* (P1)
*I was extremely well prepared and was ready to become a Community Navigator for all the good, the bad, ugly, and different. We all prepared for everything no matter what happens*. (P11)
Connections built with other Community Navigators
Being connected to the other Community Navigators across gender identities, sexual orientations and racial/ethnic identities increased a sense of belonging, resource sharing, creativity and problem solving.
*Well for me, I am a helper. I have always been an advocate for the transgender community but didn't have the opportunity to be part of a group before, so having that opportunity was even better than what I thought it would be. It was very special*. (P6)
*I think [the monthly Community Navigator meetings] were probably the most impactful for me, honestly. Connecting with other Navigators and being able to talk about things that we had experienced*. (P7)
Sustaining intervention aspects long‐term
Community Navigators sustained aspects of the intervention after implementation officially ended.
*I will continue work as a Community Navigator because it isn't a job but is something that needs to be done, even if I am not paid for it. It just needs to be done*. (P10)
*I think I will always be a Community Navigator, formally or informally, because I know something that I can always use and I will always be involved doing something to help others*. (P7)

##### Desire to serve as a community leader

Community Navigators expressed that they valued serving as leaders and as resources to address social determinants of health within their communities. Among the Community Navigators, there was a common sense of intrinsic motivation to engage, be involved with and help their communities of identity—LGBTQ communities and communities of colour. Community Navigators reported that they were drawn to the *Impact Triad* intervention because of the opportunity to gain knowledge to share within their social networks and because they recognized the importance of both promoting sexual health and addressing social determinants that can make it difficult for community members to take care of their health. Community Navigators also appreciated that they made a genuine impact, which furthered their desire to continue being leaders within their social networks and communities. They felt pride in their roles as Community Navigators, saw themselves as fulfilling a need for social support within their communities, enjoyed building leadership skills that could potentially create opportunities for future careers (e.g., public speaking skills) and demonstrated a desire to continue utilizing the knowledge and training they received postintervention.

##### Importance of being part of the communities in which one was working

Community Navigators emphasized that being members of the communities in which they worked was fundamental to trust building and successful intervention implementation. Community Navigators had shared experiences and identities and felt a sense of belonging with others in their communities and were known and seen as leaders in their communities before becoming involved with *Impact Triad*. These connections led to increased initial levels of rapport with community members within Community Navigators' social networks, facilitated the process of recruiting social network members to participate in the intervention and were helpful for further building trust to have conversations about a wide array of health‐related topics.

##### Value of having an official role

Community Navigators reported that having official titles and roles that were linked to well‐known and well‐respected institutions (i.e., Wake Forest University School of Medicine and Triad Health Project, an established AIDS service organization in Greensboro, North Carolina), and having received formal training through these institutions, provided a sense of reassurance that helped to facilitate relationship building with members of their communities. As a consequence, Navigators felt more confident in their knowledge about sexual health and social determinants and perceived that they were more trusted to talk with others within the community about sensitive health topics. In addition, Community Navigators highlighted the importance of having a badge indicating their role in the intervention and emphasized that receiving a stipend for their time furthered their sense of legitimacy and validated the work they performed to assist their communities.

##### Connections built with other Community Navigators

Community Navigators shared that being connected to the other Community Navigators across gender identities, sexual orientations and racial/ethnic identities increased a sense of belonging, resource sharing, creativity and problem solving. Community Navigators expressed that monthly meetings with the other Community Navigators and intervention coordinators were a time and place for growth because they brought together diverse experiences and perspectives and provided an opportunity to exchange ideas with one another, as well as discuss challenges and ways to overcome them. Navigators noted that it was valuable to know that other Community Navigators, including those with different gender identities, sexual orientations and/or racial/ethnic identities than their own, had found themselves in similar situations and to learn from one another. Thus, these conversations fostered an environment where Community Navigators felt welcome to express themselves and their ideas about promoting health within their communities through engagement and education. While each Community Navigator carried out helping activities independently with their social network members, the Community Navigators also worked as a team by providing social support to one another. These connections took place both in meetings and more informally in one‐on‐one interactions outside of meetings (including via social media) and continued after implementation ended.

##### Sustaining intervention aspects long‐term

Community Navigators reported hoping to continue their roles to improve sexual health and social determinants within their communities after intervention implementation officially ended. Many expressed a conviction to share the knowledge they had gained through the intervention, stressing that their social network members and other community members continued to come to them for information. Community Navigators reported continuing to engage in helping activities such as condom distribution and disseminating information through social media because the need within their communities persisted and because it felt natural to continue to draw on the training and resources provided by *Impact Triad*.

#### 3.2.2 Experiences implementing the *Impact Triad* intervention

Another set of themes related to Community Navigators' experiences implementing *Impact Triad*. These themes and selected quotations are presented in Table 4.

**Table 4:** Community Navigator experiences implementing the *Impact Triad* intervention (qualitative themes with select quotations from in‐depth interviews)

Engaging community members
Community Navigators identified effective and innovative strategies to engage community members.
*Con las actividades, al momento de juntarnos también teníamos comida, refrescos, botana. A veces aprovechábamos que había algún evento deportivo importante o programas como los Oscar. [With activities, when we gathered we would bring food, drinks, snacks. Sometimes we would take advantage of big sporting events or programs like the Oscars to get together.]* (P1)
*I posted fliers and updated social media about when there was going to be a testing event at Triad Health Project, and just let them know what events were happening and what we were doing*. (P8)
*Les di la prioridad a mis amigos a los que yo sabía que podrían estar tal vez en más riesgo, tuvieran menos información o que en conversaciones privadas me habían comentado que nunca se habían hecho la prueba del VIH. O algunos amigos que podrían no estar utilizando condones o otros métodos de prevención como PrEP. [I gave priority to my friends that I thought would be most at risk, or had less information, or in private conversations had mentioned to me that they had never been tested for HIV. Or other friends who might not be utilizing condoms or other prevention methods such as PrEP.]* (P1)
Meeting prioritized needs
Community Navigators met social network members' prioritized needs by educating and supporting them to access and use local resources.
*I helped my social network members to find some resources. For instance, most of the guys that I invited didn't know how to get condoms, how to get PrEP, or how to make an appointment to get STD testing. So that was very helpful, because every time they were asking me for more information and more condoms and things like that*. (P15)
*Some of my friends didn't even know where to get condoms or medications or that there are educational resources available. It's good to have associations with organizations like Triad Health Project or FaithAction [immigrant‐serving community organization in Greensboro, NC]. It made them interested or willing to talk more about it*. (P15)
*Getting the information out there is important because I find that a lot of people in our communities don't even know that PrEP exists*. (P6)
*We learned about continuing one's education, employment opportunities, and stuff like that. I think it's better if [social network members] hear from someone they know about the resources available for them*. (P9)
Building trust
Communicating about sexual health and addressing stigma and misinformation were difficult but overcome by building and nurturing trust.
*It's important to support each other, help people with whatever they need, have open conversations, and build trust, because when you build trust you can change and improve people's lives*. (P15)
*Yes, my friends definitely feel more comfortable now talking to me about sex, how to put a condom on correctly, and things like that. It's not awkward anymore. They feel completely comfortable talking about STDs and PrEP and some of them asked me to go with them to get tested and asked me questions about resources, and I have been able to give out materials with the information they need*. (P6)
*When [social network members] start to trust you, it's easier to talk about anything, so trust made it easier*. (P7)
Using social media
Use of social media complemented personal interaction to further facilitate dissemination of information and support.
*I was making posts about testing events and testing sites, informing [my social network members] about different STIs and also about jobs, job fairs, and places that may be hiring. Also about housing. I used Facebook, Grindr, and Jack'd*. (P4)
*I think social media helped us so a lot more people could be reached. I don't go to many places so being on social media allows me to get out in the world when I am not going to do it in person*. (P9)
*I used social media to share what I think is important and to show information and resources that I come across. I like to say from my own experiences and other people's experiences too, what people go through, so there is someone seeing it*. (P13)
*I posted on my Snapchat stories, usually about where to go and get tested and about getting tested regularly. I posted on Facebook on my timeline and created stories*. (P14)
Increasing awareness and knowledge
Intervention successes included increased awareness of prevalent health disparities and increased knowledge about sexual health and about resources to address disparities.
*I told them all about the ‘sexual pandemic’ and then they became curious. I taught the group how to wear a condom correctly, and about how to be safe or get PrEP if they need it*. (P14)
*I educated [my social network] about some of the bad statistics that often face the LGBTQ communities such as HIV rates, unemployment, and things like that that affect us*. (P6)
Challenges related to COVID‐19
The COVID‐19 pandemic hindered implementation and social media became the main strategy to reach social network members.
*It was way more difficult to continue with the communication. I guess that's the blessing of social media but, yes, [the pandemic] changed everything*. (P12)
*It made it very different because I feel certain things worked better in person, like teaching people how to put a condom on. [The pandemic] made it a little difficult*. (P6)
*[The pandemic] definitely impacted how many people I was able to reach, because I spread the message in the halls at school just talking to people and it affected my ability to do that*. (P14)
*La pandemia afectó a mi red social mucho porque nos dejamos de reunir, y la intimidad y el espíritu del grupo de estar reunidos en un solo lugar, la convivencia y la confianza que se crea en el grupo, se pierde y se vuelve más individual. [The pandemic affected my social network a lot since we stopped getting together, and the intimacy and group spirit from gathering in the same place, the atmosphere of trust that was created within the group, this was all lost and everything became more individualized.] (P1)*

##### Engaging community members

Community Navigators shared that they had identified effective and innovative strategies to engage community members. Community Navigators utilized various strategies to reach the individuals within their social networks. At the beginning of intervention implementation, Community Navigators focused their recruitment efforts on social network members who they perceived as being more vulnerable or at risk and as needing social support and emphasized to these social network members the benefits of their participation. Through this process, Community Navigators were able to reach a broad range of individuals, including engaging nongay identifying MSM by building trust to overcome concerns about having their sexual behaviours exposed. In addition, Community Navigators used creative ways to share information about health and local resources through social media posts and by making in‐person events and gatherings as welcoming and comfortable environments as possible for social network members.

##### Meeting prioritized needs

Community Navigators reported meeting social network members' prioritized needs by educating and supporting them to access and use local resources. Community Navigators noted that their role provided them with insight that allowed them to pinpoint needs and priorities within the community. Community Navigators were able to effectively link social network members with the resources they needed, which helped social network members stay engaged in the intervention. Through communication and interaction with social network members, Community Navigators identified and filled knowledge gaps and guided social network members to resources that would meet their needs related to social determinants (e.g., employment and education) and sexual health (e.g., how to access condoms and PrEP and how to make an appointment for STI or HIV screening).

##### Building trust

Community Navigators emphasized that communicating about sexual health and addressing stigma and misinformation were difficult but overcome by building and nurturing trust. Many of the Community Navigators reported initial challenges in communication with social network members related to sensitive topics due to stigma and discomfort. Though Community Navigators had their social network members' trust more generally, they had to work on building further trust specifically around sexual health. To surpass this barrier, Community Navigators fostered an open and safe environment and worked to normalize talking about sexual health. To make social network members feel more comfortable, Community Navigators eased into conversations about sexual health, first starting with a topic that was less sensitive or emphasizing common experiences that the Community Navigators and social network members shared, including feelings of stigma. Social network members opened up to the Community Navigators and to one another over time, and conversations about sexual health happened more naturally over the course of intervention implementation as Community Navigators continued to promote a sense of community within their social networks.

##### Using social media

Community Navigators described using social media to complement personal interaction to further facilitate dissemination of information and support. Community Navigators reported that personal interactions were an important part of the intervention because they provided a more personable approach, particularly during the early stages of implementation and when recruiting individuals within their social networks to participate. Social media was also an essential tool utilized by Community Navigators throughout the course of the intervention in addition to these personal interactions, and after the COVID‐19 pandemic began many Community Navigators adapted their interactions to rely solely on social media. Community Navigators posted on their social media accounts about resources or community events, and these posts benefited not only their immediate social network but also extended their reach to their social network members' larger networks and individuals whom they would not have interacted with otherwise. Expanding reach in these ways had a dual impact on Community Navigators' work by strengthening both their confidence as Community Navigators and their rapport within the community. In addition, Community Navigators noted other advantages of social media, including that it was a direct and instant form of communication between Community Navigators and social network members and, in some cases, made conversations about sensitive topics easier and more comfortable than in face‐to‐face communication.

##### Increasing awareness and knowledge

Community Navigators considered important intervention successes to include increased awareness of prevalent health disparities and increased knowledge about sexual health and about resources to address disparities. Community Navigators expressed that, among their social networks, there was a general lack of awareness of how young GBMSM and transgender women of colour, and particularly those in the US South, were disproportionately affected by STIs and HIV and by social determinants such as unemployment. When educating social network members about these disparities many Community Navigators reported witnessing a ‘light bulb’ moment where social network members internalized information about sexual health and local employment and education resources.

Community Navigators also successfully promoted broader definitions of sexual health, noting that within their communities sexual health had typically been equated with condom use or abstinence only. Through participating in *Impact Triad*, Community Navigators and members of their social networks came to understand sexual health as also involving screening for STIs and HIV, access to treatment and other prevention strategies such as PrEP.

##### Challenges related to COVID‐19

Community Navigators reported that the COVID‐19 pandemic hindered implementation and impacted Community Navigators in various ways. Because of the need for physical distancing, social media became the primary way the Community Navigators communicated with their social network members. Through social media Community Navigators maintained contact with their social networks, but Community Navigators expressed that members of their social networks felt isolated from one another. The connectedness Community Navigators fostered within their social network groups was reduced by the pandemic. In‐person interactions were postponed, and the closeness and ‘spirit’ or morale of the groups were negatively affected. Furthermore, Community Navigators faced additional challenges teaching social network members about certain sexual health topics such as how to correctly use condoms when they could not carry out these activities in person. However, Community Navigators were resilient and adapted quickly to utilizing social media to continue to maintain relationships with participants and engage and teach them.

#### 3.2.3 Lessons learned for future community navigation interventions

Finally, Community Navigators highlighted learnings to inform future similar interventions. Related themes and selected quotations are presented in Table 5.

**Table 5:** Lessons learned for future community navigation interventions (qualitative themes with select quotations from in‐depth interviews)

Facilitating access to broader resources
The *Impact Triad* intervention should be expanded to facilitate access to a wider range of resources.
*I think financial aspects, access to education, resources, transportation, and education are all important*. (P5)
*I think we should work more on housing, because I have learned about other programs that are available for housing*. (P4)
*Without access to food, it will affect your mental health and your physical health. It's a real problem, and then what you are willing to do to get food or what you are willing to do to get housing can affect your future. The problem is disproportionately bigger [among GBMSM and transgender women of color] than other populations*. (P13)
Building additional skills
Community Navigator training should include activities to develop a more in‐depth and diverse skillset, including more practice communicating about sensitive topics and promoting healthy coping strategies.
*It would be good to learn more about how to talk or how to deal with people who use drugs and also how to talk to people who are newly diagnosed with HIV*. (P11)
*It would help to have training about having difficult conversations and how to be honest with people without making them feel that you are judging them*. (P7)
*I think we need to do more with trauma and mental health, honestly. A good way to help sexual health is to talk about trauma because we live in a society that bombards us with traumatic things all the time*. (P13)
Increasing interactions among Community Navigators
Future iterations of *Impact Triad* should foster more interaction among Community Navigators.
*Sería bueno poder reunirnos con navegantes por años de otros proyectos que nos ayuden a buscar nuevas oportunidades y conocimiento para los navegantes. [It would be good to get together with Navigators that have worked for years on other projects who could help us find new opportunities and provide advice for new Navigators.]* (P2)
*As Community Navigators, we had our meetings every month, and we talked about any challenges, shared our thoughts, and provided some feedback. It was really friendly, to be honest. But I would like to have more interactions with the whole group instead of just once a month*. (P15)

##### Facilitating access to broader resources

Community Navigators reported that the *Impact Triad* intervention should be expanded to facilitate access to a wider range of resources. Throughout intervention implementation, Community Navigators heard from their social network members that it would be beneficial to receive information about additional resources related to social determinants of health, such as transportation, food insecurity, financial literacy, housing and substance use, which also contribute to sexual risk. Other barriers such as access to health care more generally, including insurance coverage and mental health care, were also suggested for inclusion as outcomes in future iterations of *Impact Triad*. Community Navigators noted that young GBMSM and transgender women of colour in different geographical areas experienced different needs, highlighting the importance of expanding the resources shared through the *Impact Triad* intervention. Community Navigators also suggested potentially adapting the intervention to reach broader communities, such as non‐LGBTQ communities, or to meet the unique needs of specific subgroups such as bisexual men.

##### Building additional skills

Community Navigators indicated that future trainings should include activities to develop a more in‐depth and diverse skillset, including more practice communicating about sensitive topics and promoting healthy coping strategies. Community Navigators shared that their training prepared them well for interacting within their social networks but that they experienced challenges in a few areas. Community Navigators felt they would benefit from more skill‐building, particularly through role‐playing, that emphasized effective ways to talk about sensitive and complex topics, such as how to engage in dialogue around a recent positive HIV diagnosis. Many Community Navigators noted that they found it difficult to have conversations about mental health, healthy coping strategies and how to overcome fear among social network members to access services when needed. They noted that more training would be useful to build their skills and increase their self‐efficacy to have these difficult conversations.

##### Increasing interactions among Community Navigators

Community Navigators expressed that future iterations of *Impact Triad* should foster more interaction among Community Navigators. Community Navigators reported that they formed a support network with each other during the monthly meetings with one another, during which they shared ideas, challenges and successes. Many expressed a desire for a greater frequency of these meetings to discuss barriers and receive feedback. To further increase interaction and learning opportunities among Community Navigators, Community Navigators suggested that in future iterations of the intervention, former Community Navigators could be invited to mentor newer Community Navigators and share their experiences, thus expanding the network of trained Community Navigators and building on real‐life experiences of serving as Community Navigators.

## 4 DISCUSSION

Analysis of in‐depth interviews with the *Impact Triad* Community Navigators both highlighted the ways that intersecting factors magnify health risks among GBMSM and transgender women of colour and illustrated the strengths of the intervention in raising awareness and disseminating information pertaining to sexual health and social determinants of health. One of the greatest contributing factors to the success of the *Impact Triad* intervention was the Community Navigators' ability to forge and nurture connections and relationships with members of their social networks and then build on this foundation to discuss sensitive topics with confidence and increase awareness, knowledge and use of local resources among these social network members. This finding aligns with existing literature that points to the potential of community‐based peer navigators to reach marginalized communities affected by health disparities and to connect community members to services because community‐based peer navigators understand community needs and strengths on a personal level and reflect the ways that members of their communities communicate and interact.^29^, ^30^, ^31^ The desire expressed by Community Navigators to sustain their work to continue to improve the overall health of their communities further illustrates the potential impact of the intervention.

Another factor playing a role in the success of *Impact Triad* was the Community Navigators' flexibility and ability to pivot and adapt to the unprecedented circumstances brought by the onset of the COVID‐19 pandemic. Initially, Community Navigators' interactions with members of their social networks included in‐person communication, which suddenly became no longer possible during the early phases of the pandemic. Adapting to the pandemic led to increased integration and utilization of social media platforms to cultivate and strengthen relationships. Shifting to an entirely virtual implementation of *Impact Triad* modified previous perceptions of how to connect with individuals and was consistent with documented changes in the ways in which networks provide social support in the context of COVID‐19 with shifts away from physical interaction and toward the use of technology.^32^ In some ways, these changes increased accessibility, allowing Community Navigators to overcome logistical barriers to engagement based on locations, schedules and work demands; these learnings could also greatly benefit research efforts in rural and other more isolated communities. However, restrictions due to COVID‐19 did affect camaraderie among Community Navigators and members of their social networks. Community Navigators noted that there was a personal aspect of face‐to‐face contact that social media could not replicate. Nonetheless, our findings demonstrate that the versatility of social media is a major asset in conducting intervention research. Other intervention studies in recent years have reported making similar adaptations due to COVID‐19,^33^, ^34^, ^35^ and future studies should continue to explore ways to achieve the connection typically built and reinforced through in‐person interaction in a virtual setting.

Interviews also identified implications for future iterations of the *Impact Triad* intervention to seek to further address and improve the quality of life among young GBMSM and transgender women of colour. A common thread across Community Navigators' experiences involved the intricate ways that interconnected social determinants impact health. To have a more profound impact, future efforts can expand the domains of resources and education provided by Community Navigators by incorporating into Community Navigator training information on how to address needs such as housing, food insecurity, transportation and others, in addition to employment, education, social support and discrimination. Furthermore, leveraging the experiences of existing Community Navigators to provide mentorship and support to future Community Navigators can lead to an increasingly cohesive group dynamic and greater community capacity. For example, future Community Navigator trainings and monthly meetings could include components in which Community Navigators from previous cohorts are present to share their experiences, offer ‘tips’, and answer questions from new Community Navigators.

### 4.1 Limitations

It is possible that social desirability bias could have influenced Community Navigators' responses to interview items. Given that interview data were collected and analysed by members of our CBPR partnership that had developed and implemented the *Impact Triad* intervention, Community Navigators may have felt inclined to describe *Impact Triad* and their experiences with the intervention in ways that were positive and less comfortable sharing constructive criticism or difficulties they had faced as part of *Impact Triad*. We tried to mitigate this possibility and encourage open and honest responses by ensuring confidentiality and emphasizing that there were no right or wrong answers and that Community Navigators were experts in their own experiences. Furthermore, interviews were conducted by CBPR partnership members who were not directly responsible for training and supporting the Community Navigators.

## 5 CONCLUSIONS

These interviews identified important learnings about serving as Community Navigators and implementing *Impact Triad* and provided information to guide future efforts to address STI and HIV disparities and critical social determinants of health through community‐based peer navigation. Further research is warranted given that community‐based peer navigation remains an understudied yet promising approach to health promotion and disease prevention among communities and populations experiencing health disparities.

## CONFLICT OF INTEREST

The authors declare no conflict of interest.

## Data Availability

The data that support the findings of this study are available from the corresponding author upon reasonable request.
